# Supplementary material for: Ultrafast population coding and axo-somatic compartmentalization
Source: PLoS Comput Biol. 2022 Jan 18;18(1):e1009775. doi: 10.1371/journal.pcbi.1009775 (PMC8797191; doi:10.1371/journal.pcbi.1009775)
Supplement: S1 Fig — AP waveforms are generated with constant inputs reproducing 5 Hz firing rate. Axonal and somatic voltages are reset to the resting potential when axonal voltages reach 0 mV. (PDF) [file pcbi.1009775.s001.pdf]

Supporting figures

In S Fig 1, we compare the axonal and somatic AP waveforms for various AP initiation sites. These AP waveforms are generated with constant current of amplitudes that cause 5 Hz firing rate for all different values of x_{Na}. Axonal and somatic voltages are reset to resting potential when the voltage in the axon reaches 0 mV. Therefore, the peak voltage in the soma is smaller as x_{Na} increases.

The intrinsic AP initiation dynamics in the axon is more voltage sensitive when x_{Na} is larger, therefore the corresponding axonal AP waveform is slightly sharper. Seen at the soma, due to voltage decoupling, the somatic voltage rises more rapidly.

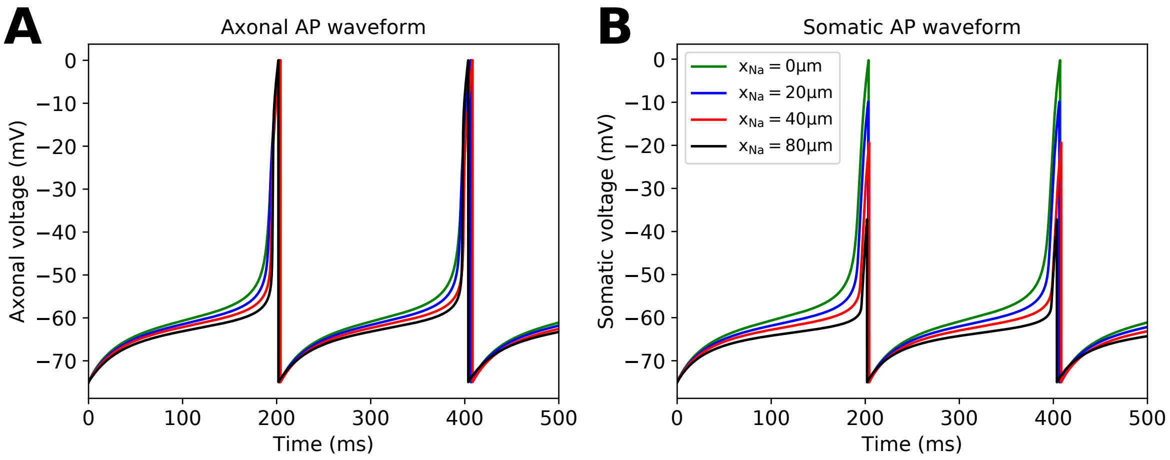

Supplementary Figure 1: **Axonal and somatic AP waveforms with various AP initiation sites.** AP waveforms are generated with constant inputs reproducing 5 Hz firing rate. Axonal and somatic voltages are reset to the resting potential when axonal voltages reach 0 mV.
