# Supplementary material for: Ultrafast population coding and axo-somatic compartmentalization
Source: PLoS Comput Biol. 2022 Jan 18;18(1):e1009775. doi: 10.1371/journal.pcbi.1009775 (PMC8797191; doi:10.1371/journal.pcbi.1009775)
Supplement: S2 Fig — A, C, E For each ka, axonal voltage derivatives of three model variants aligned at (0 mV, 0 mV/ms). B, D, F Dynamic gain functions calculated with the firing rate fixed at 5 Hz, CV of ISI fixed at 0.85. τ is set to 5 and 50 ms for each model variant. (PDF) [file pcbi.1009775.s002.pdf]

AP initiation dynamics is substantially more voltage sensitive when k_a is reduced from 6 mV (S Fig 2A) to 1 mV (C), then to 0.1 mV (E). For $k_a = 6$ mV, axonal voltage derivatives increase for less than 2 mV/ms within 10 mV (A). While for $k_a = 1$ mV, the axonal voltage derivatives increase for more than 10 mV/ms within 5 mV from their local minima (C). When $k_a = 0.1$ mV, the AP initiation dynamics is like a step function for all three model variants (E). Fixing the firing rate at 5 Hz and CV of ISI at 0.85, the dynamic gain functions display higher bandwidth, and larger Brunel effect (τ increased from 5 ms to 50 ms), when k_a is smaller (B, D, E).

Increasing x_{Na}, the spatial separation between AP initiation site and soma, the axonal voltage derivative increases slightly faster when $k_a = 6$ mV. The dynamic gain in the high frequency region is also enhanced, reflecting the acceleration of AP initiation speed. In contrast, when the AP initiation dynamics is extremely voltage sensitive ($k_a = 0.1$ mV), x_{Na} has limited impact on AP initiation speed. And the corresponding difference in dynamic gain is mainly due to the electrotonic filtering (Fig 3 in main text). Compared to $k_a = 6$ mV and 0.1 mV, $k_a = 1$ mV is an intermediate stage where the impact of AP initiation site on dynamic gain functions changes with τ. For $\tau = 5$ ms, increasing x_{Na} reduces the dynamic gain in the high frequency region, similar to $k_a = 0.1$ mV (F). While for $\tau = 50$ ms, increasing x_{Na} enhances the dynamic gain between 20 to 100 Hz is similar to $k_a = 6$ mV (B). At higher frequencies, the gain curve decays faster when x_{Na} is larger.

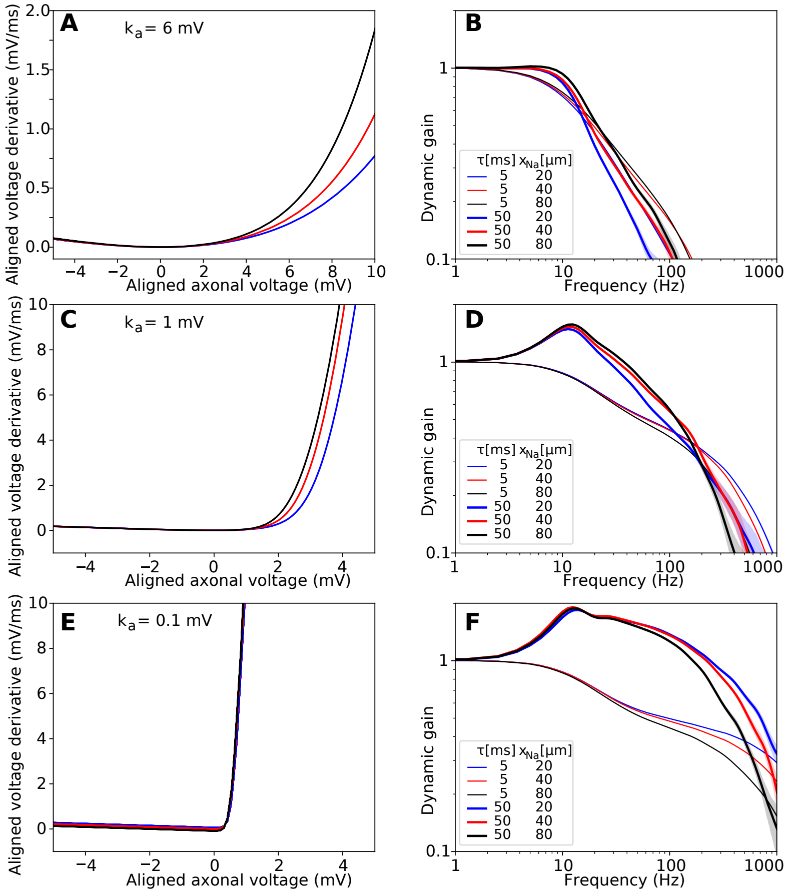

Supplementary Figure 2: **Impact of AP initiation site on AP initiation dynamics and dynamic gain functions with various k_a. A, C, E** For each k_a, axonal voltage derivatives of three model variants aligned at (0 mV, 0 mV/ms). **B, D, F** Dynamic gain functions calculated with the firing rate fixed at 5 Hz, CV of ISI fixed at 0.85. τ is set to 5 and 50 ms for each model variant.
