# Supplementary material for: Ultrafast population coding and axo-somatic compartmentalization
Source: PLoS Comput Biol. 2022 Jan 18;18(1):e1009775. doi: 10.1371/journal.pcbi.1009775 (PMC8797191; doi:10.1371/journal.pcbi.1009775)
Supplement: S3 Fig — xNa is fixed at 40 μm. ka is set to 6 mV, 1 mV and 0.1 mV in A, B and C respectively. Increasing τ from 2 ms to 50 ms, the enhancement of dynamic gain in the high-frequency region is larger when ka is smaller. The same working point was used for all simulations (CVISI = 0.85, ν = 5 Hz). (PDF) [file pcbi.1009775.s003.pdf]

In S Fig 3, we also examined the impact of τ on dynamic gain functions with more values. Increasing from 2 ms to 50 ms, the enhancement of dynamic gain at intermediate frequencies is larger when k_a is smaller.

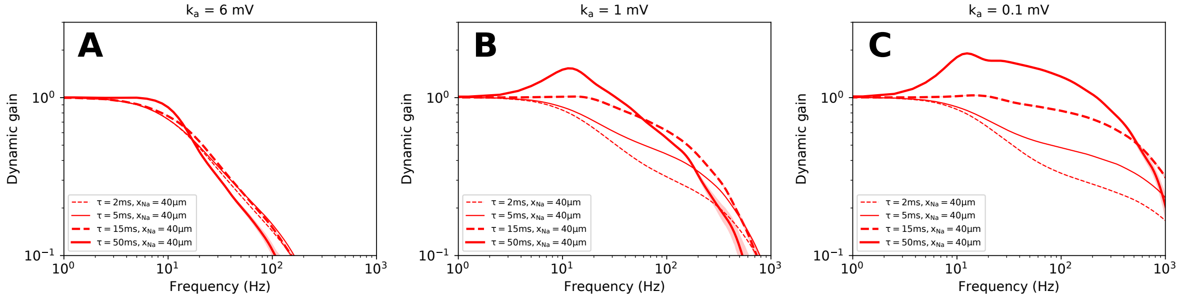

Supplementary Figure 3: **Brunel effect is larger when AP initiation dynamics is more voltage sensitive.** x_{Na} is fixed at 40 μm. k_a is set to 6 mV, 1 mV and 0.1 mV in A, B and C respectively. Increasing τ from 2 ms to 50 ms, the enhancement of dynamic gain in the high frequency region is larger when k_a is smaller. The same working point was used for all simulations ($\mathrm{CV}_{\mathrm{ISI}} = 0.85$, $\nu = 5$ Hz).
