# Supplementary material for: Ultrafast population coding and axo-somatic compartmentalization
Source: PLoS Comput Biol. 2022 Jan 18;18(1):e1009775. doi: 10.1371/journal.pcbi.1009775 (PMC8797191; doi:10.1371/journal.pcbi.1009775)
Supplement: S4 Fig — To simulate myelination of the axonal section, starting 60 μm from the soma, the specific membrane conductance was decreased 50 fold to 6.6*10−7S/cm2. The specific membrane capacitance was decreased 37.5 fold to 0.02μF/cm2. xNa is 40μm. ka is 6mV. The firing rate is fixed at 5 Hz, and CV of ISI is 0.85. With a myelinated axon, the threshold is lower and therefore, AP initiation dynamics is slower (A and C). Bandwidth of the dynamic gain functions is hardly changed compared to those of the Brette’s original model (B and D). (PDF) [file pcbi.1009775.s004.pdf]

We also examined the impact of a myelinated axon on AP initiation dynamics and population encoding (S Fig 4). The first 60 μm of the axon were left unchanged, beyond that, the specific membrane conductance was decreased 50 fold to $6.6 * 10^{-7}$S/cm^2. The specific membrane capacitance was decreased 37.5 fold to 0.02μF/cm^2. All other model parameters were kept constant. With the AP initiation site 40 μm away from the soma, introducing a myelinated axon leads to slow AP initiation dynamics as compared to the original model (A and C). Fixing the firing rate at 5 Hz and the CV of ISI at 0.85, we calculated the dynamic gain functions of Brette's model with a myelinated axon at 4 τ values (2, 5, 15, 50 ms). With a myelinated axon, the dynamic gain functions have low cutoff frequencies and decay faster compared to the original model (B and D). Increasing τ has only a small effect on the cut-off, which fails to reproduce the Brunel effect as observed experimentally.

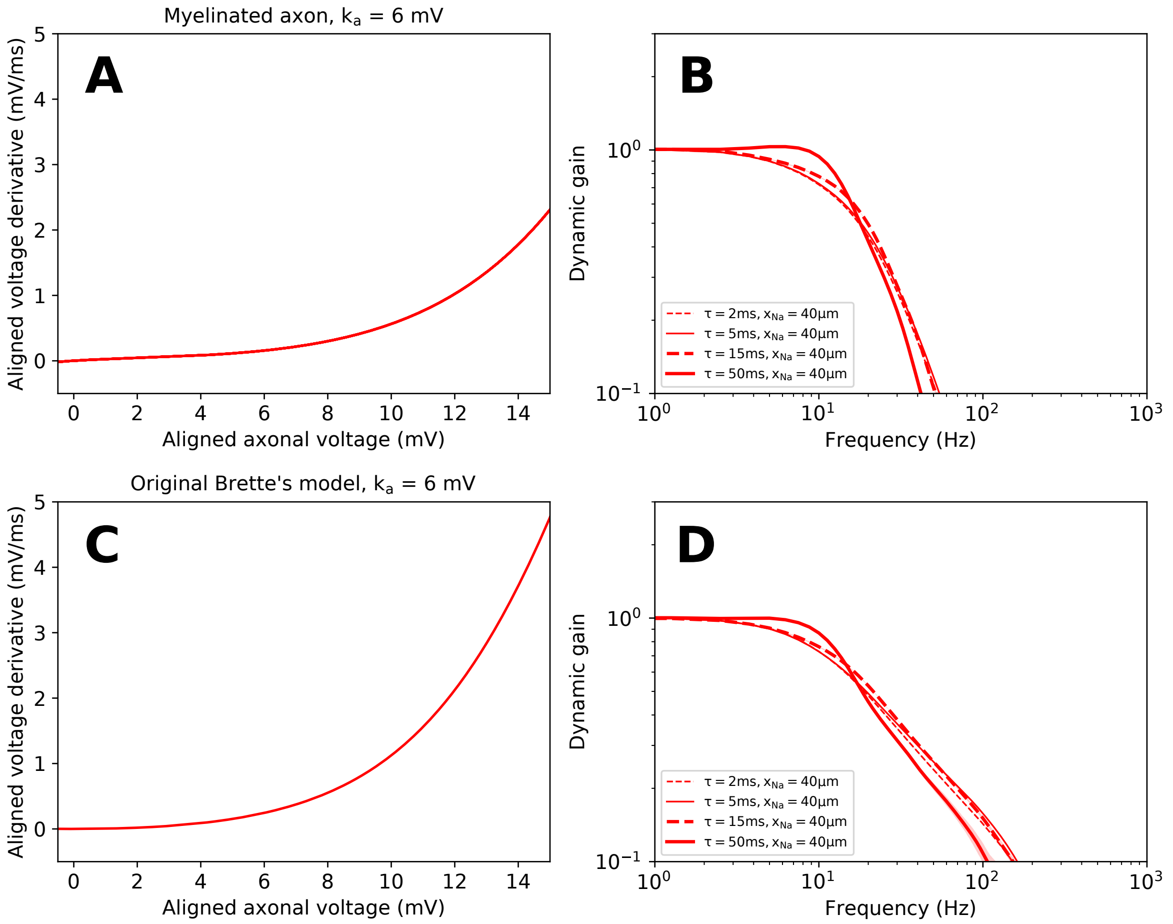

Supplementary Figure 4: **Myelination of the axon does not enhance the encoding bandwidth.** To simulate myelination of the axonal section, starting 60 μm from the soma, the specific membrane conductance was decreased 50 fold to $6.6 * 10^{-7}$S/cm^2. The specific membrane capacitance was decreased 37.5 fold to 0.02μF/cm^2. x_{Na} is 40 μm. k_a is 6 mV. The firing rate is fixed at 5 Hz, and CV of ISI is 0.85. With a myelinated axon, the threshold is lower and therefore, AP initiation dynamics is slower (A and C). Bandwidth of the dynamic gain functions is hardly changed compared to those of the Brette's original model (B and D).
