# Supplementary material for: Ultrafast population coding and axo-somatic compartmentalization
Source: PLoS Comput Biol. 2022 Jan 18;18(1):e1009775. doi: 10.1371/journal.pcbi.1009775 (PMC8797191; doi:10.1371/journal.pcbi.1009775)
Supplement: Attachment — Submitted filename: Review of Zhang et al.pdf [file pcbi.1009775.s005.pdf]

Review of Zhang et al., "Ultrafast population coding and axo-somatic compartmentalization"

This paper deals with the fact that cortical neurons, unlike single-compartment Hodgkin-Huxley models, can transmit very high frequency signals, as assessed for example by transfer functions measured with noise current. Specifically, it examines whether the simplified model of Brette (2013, Fig. 1-2), based on distal axonal initiation, can account for it, and concludes that it cannot. The paper concludes that resistive coupling theory of spike initiation, introduced and illustrated with that model, cannot account for the phenomenon.

Regarding this model, the conclusion seems justified. However, one must not equate theory and one particular model (there have been a few more elaborate models, e.g. in Goethals & Brette 2020, cited, or even in the 2013 paper). Resistive coupling theory examines spike initiation in the idealized setting where the soma clamps the base of the axon (by virtue of the difference in size), and all dynamical phenomena in the axon are considered instantaneous. This corresponds to the stationary situation of a somatic voltage-clamp, as shown here in Fig. 1. In the ideal setting of the theory, spike initiation is equivalent to a hard threshold, and therefore will show transmission of high frequencies essentially like an integrate-and-fire model. (As a side note: the theory is primarily about spike initiation, and not so much about spike shape or the lateral current.)

Particular models are meant to test whether the theory is robust to legitimate deviations from its assumptions. For example, the simple model includes axonal filtering and Nav channel activation dynamics, which are neglected in the theory but present in real neurons. But the model also deviates from theory in ways which might not be legitimate. In particular, the model has a soma but no dendrites, and although the soma might seem large (much larger than a typical pyramidal cell soma), the somatodendritic compartment is still much smaller than that of a typical pyramidal neuron (the capacitance of a 50 μm spherical membrane is about 60 pF, much smaller than the input capacitance of a pyramidal cell). This implies that, in the model, the soma does not clamp the base of the axon as much as in real neurons.

This appears to be a key point to determine whether the neuron operates in the resistive coupling regime (see the first part of Goethals & Brette, 2020; the issue was also examined for somatic onset rapidness in Telenczuk et al., 2017, Fig. 10). The simplest way to incorporate this is to add a dendrite as in Goethals & Brette (2020).

Thus, I would recommend the authors to run their analysis on the simple biophysical model described in Goethals & Brette (2020), which could be further simplified by removing Nav inactivation and Kv channels; in other words, to add a long 6 μm dendrite. Since Fig. 10B shows that increasing the size of the soma substantially increases high-frequency transmission, it seems plausible that simply adding a dendrite will make a large difference. Note also that the total Nav conductance is much higher than in the simple model from Brette (2013) (a model with higher conductance is also used in Fig. 3 of that paper, and this is necessary to get normal somatic spikes). It might be necessary to spread Nav channels over an extended AIS in order to get a normal somatic spike, as described in Goethals & Brette (2020), but maybe it is not crucial for the present question.

Another aspect by which the simple model deviates from the theory is that the axonal membrane does not respond instantaneously, because of its capacitance. This is legitimate, but the simple model overestimates the phenomenon because pyramidal cell axons are normally myelinated. This could be incorporated by increasing the membrane capacitance beyond ~50 μm. In brief, the theory predicts that in the idealized setting, the neuron responds like an IF model, but in practice the high-frequency behavior will be limited by the limiting factor, be it axonal capacitance, Nav activation dynamics or total Nav conductance (which determines axonal speed of rise).

Minor remarks

- p4, I didn't understand what was meant by "In this case, the lateral current from axon to soma not only slows axonal depolarization"; the lateral current is also there in voltage-clamp.
- Fig. 1B, there is a typo in the caption.

- p6, It is not really the lateral current that slows down the dynamics. The time constant of the axonal compartment is ~R_a.C, where R_a is the coupling resistance between soma and initiation site. So when the initiation site is moved away, this time constant increases proportionally. The filtering is due to the charging time of the axonal membrane capacitance (in other words, a membrane current).
- p7, Fig. 3. The title should rather be something like "Impact of initiation site distance…". The results shown here do not directly demonstrate a causal role of the lateral current. In particular, the forward filtering between soma and AIS are due to membrane currents (capacitive and leak).
- Fig. 5. I am a bit puzzled by this figure: it seems that increasing gNa lowers the cut-off frequency, which is unexpected and seems contradictory with the results shown in Fig. 4.
- p11, "the axonal voltage and somatic voltage are nearly identical until V1/2 is reached": indeed, theory predicts the difference is ~ka at threshold (Brette, 2013). But patch clamp studies report ~8 mV (Kole & Stuart, 2008), so this seems rather to contradict the low ka hypothesis.
- p18, gNa = 5.23 . 10^3 S. There must be an error as this is about 10 billion times larger than expected.
- p20, equation (4). It seems to me that the mean rate should be subtracted on the right hand side.
- There are formatting issues with references (brackets are missing).
- A discussion point: patch clamp studies tend to report values of ka ~6 mV, but that corresponds to Boltzmann fits over a large voltage range. When fitted over the initiation zone, it tends to be a bit smaller (4-5 mV). This is discussed for example in Platkiewicz & Brette (2010; Fig. 10).
- It would be helpful to see the code.
