# Supplementary material for: Ultrafast population coding and axo-somatic compartmentalization
Source: PLoS Comput Biol. 2022 Jan 18;18(1):e1009775. doi: 10.1371/journal.pcbi.1009775 (PMC8797191; doi:10.1371/journal.pcbi.1009775)
Supplement: Attachment — Submitted filename: Response to Reviewers.docx [file pcbi.1009775.s006.docx]

*Reviewer #1*

This manuscript appears to be a sound study of the dynamic gain function of a neuron and the role of the axon initial segment. Brette and collaborators showed that the presence of axon initial segment results in a sharp spike initiation at the soma. The present manuscript shows that this does not imply high frequency spike rate transmission and that the latter depends of the voltage sensitivity of sodium channels. I think this is a welcome clarification.

My only reservation about the manuscript is that although it uses a reduced model, results are only obtained by numerical simulations. It would have been nice to support some of the conclusions by analytical results. I wonder whether this would be possible perhaps in an even simpler model, like a two-compartment soma-AIS model.

Thank you for your appreciation of the manuscript.

Following the approach of Romain Brette, we have formulated a simple two-compartment reduced model and attempted an analytical solution. An approximate solution is possible under time-scale separation, with voltage dynamics much slower than the ion channel dynamics. However, as the comparison of the voltage-clamped case and the dynamic case in figures 1 and 7 demonstrate, the time scales are not separated; when the soma is not voltage-clamped, the bifurcation does not occur.

As a consequence, our attempts at an analytical solution have not been useful to describe the initiation dynamics.

*Reviewer #2*

Thank you very much for your critical questions! They induced us to include methodological considerations in the second discussion paragraph. A more detailed answer to each specific point is given below your comments.

The model is "simple" and not realistic. With abundance of multicompartmental, anatomically precise, biophysical models of cortical, hippocampal, and cerebellar neurons, I would have expected Authors to extend their reasoning and test their predictions and analysis also on more detailed models. The usefulness of simplified description is of course recognized but as numerical simulations and model data sharing are increasingly easy to perform nowadays, it seems that a more robust story could have been constructed.

Thank you for your comment. It expresses the hope that studying more and more examples of neuronal dynamic gain functions will elucidate the origin of the high bandwidth. Other groups have taken this approach (Verbist et al. PLoS Comp Biol 2020) and showed interesting phenomenologies, albeit without mechanical explanations. We studied the dynamic gain function of multi-compartment neurons with multiple ion channels extensively. Some results are published already (Lazarov et al. Sci Advance 2018); others are in preparation.

From these studies, we identified one fundamental complication in the use of such complex models for dynamic gain studies: several parameters influence the dynamic gain shape, but these parameters cannot be changed independently of each other, because together they also determine excitability, working point (firing pattern) and AP shape. Due to these intricate interdependencies, biophysically realistic models do not offer a simple, single-track approach to analyze dynamic gain *parameter variation*. Instead, a dynamic gain *decomposition* can be used. We started to publish our results in this field (Merino et al. accepted for publication by PNAS), and are currently preparing a publication on this topic with simulated and experimental data. However, the first step is to address the fundamental constraints of necessary and sufficient conditions for high bandwidth, ultra-fast encoding. This is achieved here in a model, which is reduced to the minimal set of parameters that suffice to reproduce the critical influences discussed in the field. We have no reason to believe that the reduction in complexity has restricted the possible phenomenology because a voltage-dependent, intrinsic action potential initiation dynamics is still present.

Another point on the realism concerns the biophysical description of the action potential. The model considered by the Authors cannot produce "naturally" a train of action potentials. In fact, the lack of sodium channels inactivation and of repolarizing currents, as explained in the Methods, is replaced by a hard "reset" of the membrane potential. While I understand that reducing the number of free parameters simplifies their systematic explorations, especially in the context of analytical exploration of a set of non-linear equations (e.g. at the steady-state), I fail to gain a convincing proof of the equivalence of the model response properties with and without the additional biophysical models. Once more, as Authors have performed numerical explorations, they might have provided an equivalence of "full" and "reduced" models.

Thank you for your comment. Indeed, Romain Brette’s model features a biophysically motivated action potential initiation but not realistic termination. However, it has been shown in our previous works that AP hyperpolarization does not imbue a model with the ability to realize high bandwidth encoding.

In Huang et al. PLoS ONE 2012 we examined the dynamic gain function of a Wang-Buzsáki model. The neuron model is a conductance-based model with realistic spike initiation and termination dynamics. With a conventional voltage sensitivity of the sodium activation function, the neuron model does not realize high bandwidth encoding, similar to that of EIF model shown in Fourcaud-Trocmé et al., J. Neurosci. 2003. The model did, however, realize a higher bandwidth when the voltage dependence of the depolarizing current was increased.

Therefore, although resetting the voltage is not entirely equivalent to the conductance-based AP hyperpolarization, its major impact is a slight change of the inter-spike interval. However, that does not undermine our findings on the impact of suprathreshold dynamics on high bandwidth encoding.

Another simplification is the use of current-based synaptic inputs versus conductance inputs. I believe it might be important to first prove their equivalence, in the specific context considered by the Authors. I fail to understand the advantages and opportunity in terms of simplicity of numerical simulations, while I believe that an activity-dependent "effective membrane time constant" may add additional realism to the Authors' point.

Thank you for your comment. We had carefully considered the use of conductance vs current and disregarded it for the following reasons: the key dynamical variable is the membrane voltage *V* at the initiation site. Input provides an external source for the temporal change *dV/dt* of that variable. In that respect, the physical nature of the input is not relevant to the dynamics.

The presence of an additional conductance at the soma has some minor impact on the voltage dynamics, in as far as it determines the intrinsic time constant at the soma. However, this impact is very small at action potential initiation sites away from the soma (Chandelier cell input notwithstanding, which obviously will have a huge impact -see work from the Burrone lab).

At its core, your question is, therefore, whether a different level of somatic leak would change the dynamics. We have addressed the impact of the somatic current sink by exploring soma sizes. This includes unphysiologically large soma versions, which well overrepresent the clamping effect of even a leaky soma.

Regarding the equivalence of conductance- and current-based estimation of dynamic gain, we would like to point out that experimental studies using conductance-based input found the same fundamental behavior (bandwidth, slopes) as current based studies: i.e. Linaro et al. vs Tchumatchenko et al.

In addition to the reason given above, we would also like to point out that conductance changes on distal dendrites are effectively current sources as far as somatic dynamics is concerned. Thus, the comparison between physiological inputs (conductance changes distributed across soma and dendrites) and current injections to the soma is not as straightforward as commonly thought.

A final concern, related to the previous point, is the choice of the parameters of the OU process. If I am not mistaken, the "mu" and "sigma" should not be considered independent, as they have been. In the "conductance-driven" input recreation, I think "mu" and "sigma" will be both linked together. I understand the need of fixing the "operating point", in terms of firing frequency and coefficient of variation of the interspike interval distribution, but wonder whether the same operating point (e.g. in vivo high CV) would still be physiologically compatible with synaptic (conductance) stimulation.

Thank you for your comment. It is correct that the phase-space of current-driven membrane voltage is larger than the space of conductance-driven membrane voltage because the latter is bounded by the reversal potentials of potassium conductance (app. -85 mV) and non-specific cation conductance (app. 0 mV). Our approach has therefore searched a larger space, and we have no reason to assume the result of the paper changes under conductance input. In other words, we contend that conventional ion channel voltage sensitivity, combined with resistive axon-soma-coupling, will not result in a wide bandwidth, even if the voltage fluctuations are induced by conductance changes at the soma, rather than current injections.

It is correct that for a *single* type of synaptic input (e.g. Glutamatergic), μ and σ are coupled. However, for two inputs with different reversal potentials (non-specific cation Vrev≈0 mV AND chloride Vrev≈-80 mV for [Cl]i=6mM, [Cl]o=150 mM), the *two* conductances have independent average values. They map a two-dimensional parameter space, approximating the two degrees of freedom that we use. For membrane potential values between those reversal potentials, the overall input is the sum of a positive input, for which μ and σ are correlated, and a negative input for which μ and σ are anticorrelated. The combination of the two inputs allows for unrelated μ and σ. As above, the space that we probed here might be larger than physiological. It remains to be tested, whether a given target operating point can be reached under physiological conditions. However, for our statement, that is not a problem.

*Reviewer #3*

A more systematic exploration of how key features of the dynamic gain curve (cutoff and high frequency slope) vary with system parameters would be important.

We would like to address this request in two parts.

I) The dependency of *cut-off frequency* on model parameters is presented for varying ka, gNa, xNa, soma-size, and correlation time. In our opinion, this exhaustive characterization sets a standard for dynamic gain studies.

II) The slope of the gain curve for f—>infinity has received substantial attention in the analysis of simple models. This is understandable because (1) limits can often be calculated exactly, even when solutions at finite frequencies are approximations, and (2) limit behavior can reflect properties of the underlying dynamical system.

In numerical studies of realistic neurons, however, multiple factors impact the slope. Those are listed below, and we disentangle them in another study on models with more channels and dendrites.

The present manuscript would not benefit from an extended discussion of limiting slopes. Here we address the decay in the 10 to 100 Hz range that is present in every published type I model with conventional voltage dependence but not in real neurons. The fact that the slope at high frequencies is not -1 can have many reasons (below), that do not elucidate the origins of the decay between 10 and 100 Hz.

___________________

Factors affecting gain decay at f>1000:

1) Finite ion channel activation time constant (mentioned in discussion)
2) Finite threshold, this causes a flat gain curve at sufficiently high frequencies for any initiation mechanism
3) Limited intrinsic current. See also the answer to your question regarding figure 2B (below).

Would a spatial EIF model (Aspart, Ladenbauer and Obermayer, PLoS CB, 2016) be able to reproduce the present results? Similar to a comparison in the original Fourcaud-Trocme et al (2003) paper.

In principle, yes. We had detailed this in the discussion. For your convenience, we elaborate on those considerations here.

The effect of morphology on the *filtering of injected current* can be captured in such an approach, either analytically or numerically, for more complex morphology. We mentioned this in our discussion: “For simple morphologies, such passive effects can be understood analytically [citation Aspart].”

However, the impact of morphology on the *filtering of the intrinsic current*, the loss of axonal sodium current due to the somatic current sink, is not represented in the ansatz of Aspart et al. Therefore, the impact on the initiation speed (figure 11) would not be estimated correctly. We elaborate on this in the discussion immediately following the above sentence:

“Our analysis reveals another effect of the electrotonic structure, mediated by an altered AP initiation dynamics. An increased somatic current sink drastically reduces the depolarizing currents available at the initiation site. However, the voltage threshold for AP initiation is also strongly shifted towards the voltage at which the ion currents attain their peak voltage dependence (Fig11). These two effects have opposing impact on the activation dynamics at threshold and consequentially, the combined effects of increased somato-dendritic size can reduce the bandwidth, as in the case of high voltage sensitivity ka=0.1 mV, or increased the bandwidth, as in the case of a low voltage sensitivity ka= 6mV, similar to the waveform AP waveform change reported by [citation Eyal2014].“

The Brette paper discusses distributed sodium channels. How would that impact the model presented here?

As Romain Brette showed in later papers, the distribution of ion channels across distances far below the spatial length constant does not greatly change the behavior. Heterogeneous ion channel properties together with somatic ion channels can of course modulate the *somatic* AP waveform, but the point of our paper is that the *somatic* waveform is not tightly connected to encoding. The key determinant of encoding bandwidth here is the voltage sensitivity of the AP initiation dynamics. Distributing ion channels does not affect the effective voltage sensitivity.

What are the mean firing rates in the simulation? Make sure all parameter values are clear for each figure.

Done. We use standard criteria to determine the working points across all figures, as mentioned in the introduction. In the initial submission, we had specified these criteria once, in the Method section. We now also state them explicitly the first time dynamic gain results are presented:

“It is important to note that the model parameters do not fully determine the dynamic gain function. Instead, the working point, at which the model is studied, influences the dynamic gain function. In order to provide an unbiased comparison of different models, we always selected the current’s average and standard deviation to achieve a firing rate of 5 Hz and a CV of ISI of 0.85, unless noted otherwise. In every case, spikes from 400,000 seconds of simulation were used to calculate a dynamic gain function.”

The model should be made available in an online repository. (l. 509) lists an old NEURON version. Please make sure that the simulations also run on the recent version (8.0).

Done. The code runs without changes on NEURON 8.00 and python 3.97. The results are identical. All figures produced for the resubmission were obtained with this configuration.

Thank you for pointing out the missing reference to the public code repository. We now added it:

“All code is available at https://github.com/chenfeizhang/Brette_gwdg”

(l. 51) Reference 23 is used twice in the sentence.

Corrected, thank you for the careful reading.

(l. 127) It would be worth mentioning the type of mathematical bifurcation that is referred to (a cusp, a saddle-node?). I is important to be precise as there are many different types of bifurcations all with different properties of which "sudden changes" is only one.

We agree that such information would have been an important clarification in Brette 2013. However, here, we argue that the dynamically operating neuron, i.e., the neuron without a voltage-clamped soma, does not display a bifurcation. Therefore, we refrain from discussing the bifurcation, which is absent in the dynamic model, because we think that such a discussion might confuse the reader to believe that the bifurcation is relevant for the dynamic gain.

(Fig. 1B) It would be helpful to plot the distance $x_{Na}$ as labels into the subplots. The stable an unstable fixpoints should be marked as solid and empty dots.

Done, thank you for suggesting this improvement.

(Fig. 1C) Label the "discontinuity" with a different line style or jump. There seems to be discrepancy between the main text referring to Figure and the caption. Is this figure for constant current or increasing current (voltage or current clamp, spiking with 5Hz or not)?

Done, this section is now dotted. In addition, the legend for C is now more clearly written:

“Axonal voltage plotted against somatic voltage for two conditions with $x_\mathrm{Na}=40\mathrm{\mu m}$. Only when the somatic voltage is artificially fixed to a slowly increasing potential (dashed), a sudden jump in the axonal voltage occurs (dotted), when the voltage control of the axonal voltage is lost around $-55\mathrm{mV}$. In a dynamic situation, here a current clamp with injection of a constant current driving firing at 5 Hz, this jump disappears (solid line), the transition is more gradual.”

(l. 138) Although for a cusp bifurcation there is the possibility for hysteresis, it does not seem to be visible in Fig. 1C. Why?

Figure 1C is not aimed at characterizing the bifurcation. Instead, it replicates the original figure in Brette 2013 to demonstrate the equivalency of the model implementations. Most likely, Romain Brette chose to show the axonal voltage only during a voltage increase because the process at hand is action potential initiation. The second branch of the dynamics develops when the voltage drops. If we here included this branch, absent in the original publication, the demonstration of equivalent implementations would be undermined.

Notably, the one (continuous) line that we added to the figure, as compared to Brette 2013, demonstrates that the bifurcation is an artifact caused by the artificial condition of a fixed somatic voltage. This is another reason why, also for the resubmission, we decided to focus away from the bifurcation. It is an artifact that does not explain neuronal function.

To avoid confusion, we now removed the reference to the hysteresis from the main text because we do not display the hysteresis in figure 1.

(165) It would be worth restating the firing rate and CV values used also in this section.

Done, see above.

(l. 167) With a cutoff of 200 Hz (tau = 5ms) it seems difficult to estimate the higher frequency gain. In the frequency range of interest > 10^2 Hz the input process has little power. This should reflect in the estimation error bars of the spectral densities. Due to the large number of trials these are not visible. How where the errorbars calculated? Bootstrapping or error propagation? Why not use a faster time scale for the stimulus?

Your intuition is correct, and the situation is by a factor of 2 Pi more extreme than suggested. The cut-off frequency of Ornstein-Uhlenbeck process with 5 ms correlation time is (0.005*2*Pi)^-1= 32 Hz (for a visualization of the spectrum, see figure 1 of our recently updated bioRxiv manuscript <https://doi.org/10.1101/2019.12.19.882639).>

For a description of the error calculation, we refer to the methods section, which we reproduce here for convenience:

“For the bootstrap confidence interval, we used the 20000 individual simulations to calculate 400 $STA_{I}$ curves from 50 simulations each. We then bootstrap re-sampled the grand average $STA_{I}$ from these 400 STA estimates for 1000 times and obtained the corresponding dynamic gain curves. The 95 percent confidence intervals from these bootstraps are shown for each dynamic gain curve, typically they are smaller than the line width.”

You are correct to state that a shorter correlation time yields narrower confidence bands around the dynamic gain curve. Unfortunately, a shorter correlation time also results in a different dynamic gain curve (see figures 6 and 8).

(l. 173) Could you draw the $f^{-1}$ line into the plot Fig. 2B. The model with $x_{Na}=0$ does not show a hight-frequency scaling exponent of -1. This seems to contradict the simulation of the isopotential WB model in Fourcaud-Trocme et al (2003). What is the exponent How can this be resolved?

Done. The line for slope -1 is now added. Thank you for the suggestion.

The fact that the one model variant that should be the closest to the point neuron does deviate from the -1 slope, which you expected from a conductance-based point neuron, is indeed intriguing. There are at least two possible reasons: WB and EIF models use infinitely fast channel activation, in Brette’s model the conductance responds with an activation time constant of 100µs. This could limit the encoding at high frequencies. But obviously, for the 10-100 Hz frequency range, that is not the case because the variants with $x_{Na}$=20 or 40µm display a slope of roughly -1.

The second parameter that could limit the slope is the limited conductance of the sodium channels. From figure 5 A and C, you can appreciate that the cut-off frequency decreases with $x_{Na}$ only if the “standard” conductance of Brette’s model is used. However, for larger conductances, the cut-off frequency does not depend strongly on $x_{Na}$. This is illustrated more clearly in the following figure, which shows the families of dynamic gain curves from figure 5A $x_{Na}$ =0 and $g_{Na}$ increasing from “standard” to 20-fold. You can immediately appreciate that a limiting conductance is indeed the cause of the aberration.

The reason for this dependence on conductance is the interplay between external current and intrinsic sodium current around threshold. The expected behavior (slope -1) is obtained if the intrinsic current rises exponentially throughout the critical voltage range in which external and intrinsic current are of equal magnitude (Figure 2A). This is not the case for the “standard” conductance and $x_{Na}$=0.

```
(Fig. 2B) The number of spikes used to estimate the dynamic gain function (or the STA on which they are based?).
This number is given in the Method section. For convenience, we now also state it in the results as stated in response to your earlier question regarding firing rates.
(l. 193) How exactly is the AP shape altered? Maybe a Figure in the appendix is appropriate?
Done. We now added a supplemental figure S1 Depicting the waveforms of somatic and axonal action potentials.
(l. 195) It is unclear at this point what electrotonic filtering is. Is it the transfer impedance? Please elaborate. It is also not clear from the caption of Fig. 3.
Done. While cellular electrophysiologists predominately use the term “electrotonic filtering”, in this computational work “transfer impedance” might be the better term. We replaced the term throughout text and figures. How we determined the transfer impedance is described in the methods subsection with the heading “Linear response, dynamic gain and electrotonic filtering” (now “Linear response, dynamic gain and transfer impedance”)
(l. 210-214) Does this mean that the high-frequency dynamic gain varies non-monotonically with the position of the AP initiation site? If so, this is an interesting finding that could be highlighted.
Yes. We did highlight this in the main text as a consequence of transfer impedance.
(l. 216) Would the term axial resistivity be more appropriate?
Done. Thank you for your correction
 (Fig. 5) The 3d surface plots with additional colour coding are difficult to read. Could you use simple heatmaps instead?
Done. Figure 5 and the equally designed figure 9 were completely redone. One panel of the figures is shown here as an example.
```

(l. 266) Could you explore more than two tau values? What happens for smaller values than 5ms? Does the weal resonance remain and at which frequency?

In the supplementary document, we added one figure S3. Dynamic gain functions are calculated with four tau values (2, 5, 15, 50 ms) with three ka values (0.1, 1, 6 mV)

Weak resonance happens for large tau (50 ms). It appears because the firing pattern for long correlation times can feature substantial intervals, during which the current is above rheobase and the neuron fires rather regularly.

(l. 290) With only two values of ka it is difficult to see the general trend. Please increase the parameter set.

We have examined ka=1 mV and provided comparisons in supplementary figures 2 and 3.

(l. 300-301) Is the 1/f scaling of the dynamic gain affected by ka?

Theoretically, 1/f scaling is not affected by k_a_ in the EIF model. However, with smaller k_a_, the dynamic gain function would behave more similar to the LIF model in the low frequency region, and the 1/f decay should appear only at very high frequencies.

In multi-compartment model simulations, the dynamic gain in the very high frequency is affected by stimulus filtering, and can be contaminated by noise due to limited amount of data.

(l. 305-307) The fact that an increase in xNa decreases the dynamic gain seem to be the opposite of what was seen in Fig. 2B, yet it is observed in Fig. 3C. What is the relation between Fig. 3C and Fig. 8?

The relation between figures 2 and 3 is discussed in detail in the main text. In short, two opposing effects lead to a non-monotonic impact of xNa on initiation. 1) increasing xNa filters high frequency input components. This effect becomes stronger, once xNa reaches the frequency-dependent length constant $\lambda_{f}$ for those frequencies f that are encoded well by the initiation machinery. As long as xNa is smaller, i.e. within $\lambda_{f}$ -values for frequencies that are not well encoded, the extra contribution of stimulus filtering is hidden, because the dynamic gain values at f are already low, due to the limited encoding. 2) Increasing xNa accelerates AP initiation, and leads to the enhancement of dynamic gain. The acceleration of AP initiation speed is limited by the voltage sensitivity of the sodium current. Therefore, when xNa is very large, further increasing xNa has very limited impact on AP initiation speed, while the stimulus filtering effect would still become stronger, which reduces the dynamic gain.

In Figure 8, the voltage sensitivity of the sodium current is increased. In this situation, increasing xNa does not accelerate initiation any further, because it is already very fast. The effect of increased filtering is, therefore, dominant already at small xNa values.

(Fig. 8) The 3d surface plots with additional colour coding are difficult to read. Could you use simple heatmaps instead?

Done, together with fig 5. See above

(l. 333-334) Not clear to which voltage clamp scenario in Fig. 1 is referred to.

(l. 337-338) Why would it behave like an LIF? Would the spike onset dynamics be irrelevant?

These two questions demonstrated that we did not do a good job justifying the simulations in figure 11 (soma size impact). We, therefore, rephrased that justification:

“So far, we have not found a condition for which resistive coupling substantially impacts the cut-off frequency. We argued that the bifurcation observed for a voltage-clamped soma does not manifest during dynamic AP initiation in the unclamped neuron (Fig.1 continuous vs. dashed line). But there is a possibility to render the somatic voltage almost insensitive to lateral currents, even though it is not actively voltage-clamped: the soma size has to be increased and thereby, the somatic capacitance. We next study the impact of soma size on the dynamic gain.”

(l. 372-375) Could more extreme $x_{Na}$ cases be simulated to corroborate the claims here?

The impact of more extreme xNa case has been examined in previous sections, suing xNa = 200µm. Once the filtering dominates, further increase in xNa does not lead to a different effect. The filtering only gets stronger.

(l. 529) The prefactor to the second spatial derivatives in the cable equation (1) and (2) seems wrong. Should it be $\frac{d}{4R_a}$?

Corrected, thank you for spotting our mistake!

(l. 534) Should the continuity of the current boundary condition be $\frac{d^2V}{dx^2}$?

Corrected, thank you for spotting our mistake!

(l. 552-554) It is not completely clear what the reset conditions are.

We describe that in the methods:

“The reset is performed upon crossing the reset threshold, which is chosen such that on average reset occurs 2ms after the AP detection threshold was crossed.”

(l. 581) Eq. (4) does not have the firing rate removed from the Dirac delta train.

Corrected as shown below. Thank you for the careful reading!

Review #4

Review of Zhang et al., “Ultrafast population coding and axo-somatic

compartmentalization”

This paper deals with the fact that cortical neurons, unlike single-compartment Hodgkin-Huxley models, can transmit very high frequency signals, as assessed for example by transfer functions measured with noise current. Specifically, it examines whether the simplified model of Brette (2013, Fig. 1-2), based on distal axonal initiation, can account for it, and concludes that it cannot. The paper concludes that resistive coupling theory of spike initiation, introduced and illustrated with that model,cannot account for the phenomenon.

Regarding this model, the conclusion seems justified.

Thank you for the positive reading of our findings.

Below you argue that the studied model might not be the best realization of the theory, and therefore, the dynamic gain of the model does not resemble the dynamic gain of an integrate and fire model.

You suggest several modifications that might ameliorate the discrepancy. We answer the specific points below each item, but I would like to clarify the fundamental problem at the outset:

The dynamic gain of a neuron depends on the temporal dynamics of the intrinsic action potential initiation (shown by the data and by the analysis of Fourcaud-Trocme and colleagues 2003). Ignoring this dynamic, approximating it by instantaneous depolarization is an abstraction that ignores the critical determinant of the neuron’s performance. No realistic neuron model has an infinite dV/dt. Excessive soma size, dendrites or myelination will not change this (as our additional data show).

Resistive coupling drastically changes

*d (dV_axon_/dt) / dV_soma_.* It does little to

*d* *(dV_axon_/dt) / dV_axon_* , which we show to be a key determinant of dynamic gain bandwidth.

This is now expressed more clearly in the results part:

“However, it is important to note that the abrupt change of the voltage difference in Fig1B must not be confused with a very rapid change of the axonal voltage or a high voltage sensitivity of the change of the axonal voltage. Only the artificial construct $d(\frac{dV_{axon}}{dt})/dV_{soma}$ is very large.

The axonal phaseplot's slope $d(\frac{dV_{axon}}{dt})/dV_{axon}$ is not necessarily drastically changed at this bifurcation point.”

However, one must not equate theory and one particular model (there have been a few more elaborate models, e.g. in Goethals & Brette 2020, cited, or even in the 2013 paper). Resistive coupling theory examines spike initiation in the idealized setting where the soma clamps the base of the axon (by virtue of the difference in size), and all dynamical phenomena in the axon are considered instantaneous.This corresponds to the stationary situation of a somatic voltage-clamp, as shown here in Fig. 1. In the ideal setting of the theory, spike initiation is equivalent to a hard threshold, and therefore will show transmission of high frequencies essentially like an integrate-and-fire model.

The problem lies in the application of a steady-state theory to a dynamic case that never reaches steady-state. The abstraction of a hard threshold does not capture the essence of realistic action potential initiation. We show that even for extreme cases (2 cm wide soma), the relations in a dynamically operating axon do not resemble the idealized case of voltage-clamped soma that is slowly charged.

(As a side note: the theory is primarily about spike initiation, and not so much about spike shape or the lateral current.)

Particular models are meant to test whether the theory is robust to legitimate deviations from its assumptions. For example, the simple model includes axonal filtering and Nav channel activation dynamics, which are neglected in the theory but present in real neurons. But the model also deviates from theory in ways which might not be legitimate. In particular, the model has a soma but no dendrites, and although the soma might seem large (much larger than a typical pyramidal cell soma), the somato-dendritic compartment is still much smaller than that of a typical pyramidal neuron(the capacitance of a 50 μm spherical membrane is about 60 pF, much smaller than the input capacitance

of a pyramidal cell).This implies that, in the model,the soma does not clamp the base of the axon as much as in real neurons.

We have already addressed this point in the initial submission. Following this manuscript’s preprint release, you suggested studying the effect of much larger soma sizes. We followed your suggestion and investigated dynamic gain for soma sizes up to 2 centimeters in diameter. Such immense somata do not underestimate, but *over*estimate the current sink of a cortical neuron, yet the cut-off frequency increases only by a factor of 2. Our data show that this large soma drastically clamps the axon, not only at the base but even 40µm into the axon: the peak voltage at the initiation site is reduced by as much as 60 mV (fig11C). This clearly demonstrates that increasing the somato-dendritic sink is not the way to obtain an experimentally observed high bandwidth.

This appears to be a key point to determine whether the neuron operates in the resistive coupling regime (see the first part of Goethals & Brette, 2020; the issue was also examined for somatic onset rapidness in Telenczuk et al., 2017, Fig. 10).The simplest way to incorporate this is to add a dendrite

as in Goethals & Brette (2020).

Thus, I would recommend the authors to run their analysis on the simple biophysical model described in Goethals& Brette (2020), which could be further simplified by removing Nav inactivation and Kv channels; in other words, to add a long 6 μm dendrite. Since Fig. 10B shows that increasing the size of the soma substantially increases high-frequency transmission, it seems plausible that simply adding a dendrite will make a large difference. Note also that the total Nav conductance is much higher than in the simple model from Brette (2013)(a model with higher conductance is also used in Fig. 3 of that paper,and this is necessary to get normal somatic spikes).

Done. Thank you for pointing out the potential role of sodium conductance amplitudes. As you can see in figures 4 and 5, we included this parameter in our assessment. Even a 20 fold increase of the sodium conductance has almost no influence on dynamic gain bandwidth. For a more general analysis of the benefits of your model over a more complex model, we would like to point to our responses to reviewer 2.

It might be necessary to spread Nav channels over an extended AIS in order to get a normal somatic spike, as described in Goethals& Brette (2020), but maybe it is not crucial for the present question.

We agree that it could be attempted, but we also contend that the spread over distances smaller than the electrotonic length constant does not hold the promise of great changes, especially as the channels closer to the soma will contribute less to the initiation (Baranauskas, Nat Neurosci 2014). We, therefore, did not attempt it.

Another aspect by which the simple model deviates from the theory is that the axonal membrane does not respond instantaneously, because of its capacitance. This is legitimate, but the simple model overestimates the phenomenon because pyramidal cell axons are normally myelinated. This could be incorporated by increasing the membrane capacitance beyond ~50 μm.

Following your suggestion, we ran the simulations with a myelinated axon (starting 60µm from the soma). In the myelinated section, membrane resistance is 50 times higher, membrane capacitance 37.5 times lower (numbers taken from Eyal et al. 2014). All other model parameters are identical. The working point is in both cases 5Hz, CV=0.85. The result below shows that the cut-off frequency is essentially unchanged. This comparison is now included in the supplemental material

In brief,the theory predicts that in the idealized setting, the neuron responds like an IF model, but in practice the high-frequency behavior will be limited by the limiting factor, be it axonal capacitance,Nav activation dynamics or total Nav conductance (which determines axonal speed of rise).

Thank you for the very insightful juxtaposition of idealized theory and realistic simulations and experiments. We agree that the limited temporal response of the axonal membrane - even under resistive coupling, is the reason for the limited bandwidth. We disagree with the statement that the resistive coupling theory predicts a certain shape of the dynamic gain. We are not aware of any such result.

Minor remarks

-p4, I didn’t understand what was meant by“In this case, the lateral current from axon to soma not only slows axonal depolarization”; the lateral current is also there in voltage-clamp.

We do not claim, that the lateral current is absent in the voltage clamp case. Of course, it is present and, of course, is slows axonal depolarization. Any transmembrane sodium current that is not invested in local depolarization but flows away contributes to a slowing of the local depolarization. However, figure 1 is a plot of V_axon_ vs V_soma_ , time is only a parameter, and the temporal evolution cannot be appreciated.

Our sentence simply states that in the dynamic case “the lateral current from axon to soma not only slows axonal depolarization but accelerates the depolarization of the soma.” This small change of somatic voltage during drastic axonal depolarization causes the plot to deviate from the vertical dotted line.

-Fig. 1B, there is a typo in the caption.

Thank you for the careful reading! We corrected the two typos

-p6,It is not really the lateral current that slows down the dynamics.The time constant of the axonal compartment is ~Ra.C, where Ra is the coupling resistance between soma and initiation site. So when the initiation site is moved away, this time constant increases proportionally.The filtering is due to the charging time of the axonal membrane capacitance

(in other words, a membrane current).

We are not sure, what simplification is behind this statement, and we have trouble deriving it:

1) a long cable does not have a single time constant. Instead, the voltage at the current injection site rises as a weighted sum of many exponential functions with many time constants. The largest of these is τ_mem_=r_mem_c_mem_.

2) in your function, the time constant is zero when the initiation site approaches the soma? We expected that at that point, it should be τ_mem_.

-p7, Fig. 3.The title should rather be something like “Impact of initiation site distance...”. The

results shown here do not directly demonstrate a causal role of the lateral current. In particular, the forward filtering between soma and AIS are due to membrane currents (capacitive and leak).

Thank you for the suggestion. The title is changed accordingly.

-Fig. 5. I am a bit puzzled by this figure: it seems that increasing gNa lowers the cut-off frequency, which is unexpected and seems contradictory with the results shown in Fig. 4.

In figure5 A we show the data from figure 4B (and more). In Figure 5B, three data points are from figure 4A. Therefore, those data are entirely consistent with each other. Is it possible that you compared data for ν = 1 Hz (figure 4A with data for ν = 5 Hz (figure 5A)? Those indeed show different effects for increasing gNa.

The fact that the 5Hz data, but not the 1Hz data, show an unexpected behavior illustrates how important the choice of working points is when assessing dynamic gain functions.

-p11, “the axonal voltage and somatic voltage are nearly identical until V1/2 is reached”:

indeed, theory predicts the difference is ~ka at threshold(Brette, 2013). But patch clamp studies report ~8 mV (Kole & Stuart, 2008), so this seems rather to contradict the low ka hypothesis.

Thank you for this interesting comment. The experimental evidence is now mentioned in the discussion: “It remains an intriguing discrepancy that experimental characterizations of neuronal sodium channels report voltage sensitivities corresponding to $k_{a}$=4-6 mV \cite{platkiewicz_threshold_2010}.”.

-p18, gNa = 5.23 . 103S. There must be an error as this is about 10 billion times larger than expected.

There is a misunderstanding. We did not write 10^3, but 10^-3. However, there is indeed an oversight from our side. We had missed that *Neuron* uses different standard units for distributed ion channels (S/cm²) and for point processes (µS). We use a point process to model the sodium channels. The correct exponent is therefore -9 and not -3. We now corrected this in the manuscript.

-p20, equation (4). It seems to me that the mean rate should be subtracted on the right hand side.

Done, thank you for the careful reading! The resulting figure is reproduced above under the last comment by reviewer#3.

-There are formatting issues with references (brackets are missing).

Unfortunately, we could not locate the issues.

-A discussion point: patch clamp studies tend to report values of ka ~6 mV, but that corresponds to Boltzmann fits over a large voltage range.When fitted over the initiation zone, it tends to be a bit smaller (4-5 mV).This is discussed for example in Platkiewicz & Brette (2010; Fig. 10).

Thank you, this is added to the discussion.

“It remains an intriguing discrepancy that experimental characterizations of neuronal sodium channels report voltage sensitivities corresponding to $k_{a}$=4-6 mV \cite{platkiewicz_threshold_2010}.”

-It would be helpful to see the code.

Thank you for pointing out the missing reference to the public code repository. We now added it:

“All code is available at https://github.com/chenfeizhang/Brette_gwdg”
